# Brilliant X-rays using a Two-Stage Plasma Insertion Device

**DOI:** 10.1038/s41598-017-04124-7

**Published:** 2017-06-21

**Authors:** J. A. Holloway, P. A. Norreys, A. G. R. Thomas, R. Bartolini, R. Bingham, J. Nydell, R. M. G. M. Trines, R. Walker, M. Wing

**Affiliations:** 1 Department of Physics and Astronomy, University College London, London, WC1E 6BT United Kingdom; 2 John Adams Institute, University of Oxford, Denys Wilkinson Building, Keble Road, Oxford, OX1 3RH United Kingdom; 3 Department of Physics, University of Oxford, Parks Road, Oxford, OX1 3PU United Kingdom; 4 Central Laser Facility, STFC Rutherford Appleton Laboratory, Harwell Campus, Didcot, OX11 0QX United Kingdom; 5 Diamond Light Source, Harwell Science and Innovation Campus, Fermi Ave, Didcot, OX11 0DE United Kingdom; 6 Department of Nuclear Engineering & Radiological Sciences, University of Michigan, Ann Arbor, MI United States; 7 Department of Physics, University of Strathclyde, Strathclyde, G4 0NG United Kingdom

## Abstract

Particle accelerators have made an enormous impact in all fields of natural sciences, from elementary particle physics, to the imaging of proteins and the development of new pharmaceuticals. Modern light sources have advanced many fields by providing extraordinarily bright, short X-ray pulses. Here we present a novel numerical study, demonstrating that existing third generation light sources can significantly enhance the brightness and photon energy of their X-ray pulses by undulating their beams within plasma wakefields. This study shows that a three order of magnitude increase in X-ray brightness and over an order of magnitude increase in X-ray photon energy is achieved by passing a 3 GeV electron beam through a two-stage plasma insertion device. The production mechanism micro-bunches the electron beam and ensures the pulses are radially polarised on creation. We also demonstrate that the micro-bunched electron beam is itself an effective wakefield driver that can potentially accelerate a witness electron beam up to 6 GeV.

## Introduction

Accelerating charged particles in the wake of a beam propagating through a plasma, i.e. plasma wakefield acceleration, achieves energy gains thousands of times greater per metre than those in conventional radio frequency accelerators^1–9^. In conventional accelerators, electric fields above 100 MVm^−1^ ionise the metal cavity where the particles are accelerated, destroying the accelerating structure. In wakefield accelerators however, no such limitation exists as the plasma is already ionised and far greater electric fields can be realised.

Current light sources use magnetic fields to stimulate undulations in an electron beam leading to copious X-ray production^10^. In plasmas, however, it is the strong focusing electric fields found in wakefields that cause the beam to undulate, generating short X-ray pulses^11–18^. This is achieved by injecting a short electron beam into the correct phase of the wakefield where it experiences the strong on-axis transverse fields. Electron motion in wakefields is well understood^19, 20^. The electrons oscillate, emitting synchrotron radiation at X-ray frequencies comparable to those generated by third generation light sources. Plasma wakefield generated X-ray pulses have been demonstrated experimentally and used in phase contrast imaging^11^.

Here we study intense laser pulses^21^ and charged particle beams propagating through plasma as drivers of wakefield acceleration. The enormous ponderomotive force of the laser pulse, or space charge of the particle beam, acts to expel the background plasma electrons, leaving a region of ions, resulting in charge separation. The restoring force from the stationary background ions acts to pull them back to their equilibrium position where they overshoot, forming a repeating accelerating structure with large amplitude longitudinal and transverse electric fields. Laser-driven wakefield experiments have demonstrated electric fields >100 GVm^−1^ over short distances, typically millimetres, accelerating electrons to over a GeV in energy^1^. Charged particle-driven wakefields achieve lower fields experimentally, but sustain them over longer distances, due in part to the higher energy stored in a particle beam. For example, a 42 GeV electron beam at the Stanford Linear Accelerator has been used to drive a wakefield^2^ that accelerated a portion of the 42 GeV electrons to 85 GeV over 0.85 m, representing an average experienced accelerating field of 52 GVm^−1^.

In the novel scheme presented here, the advantages of both laser and particle-driven wakefields are utilised. A laser driven wakefield modulates a particle beam, enabling it to drive a wakefield. To drive a wakefield within a plasma effectively, a drive beam needs to have a length comparable to the plasma wavelength. Conventional particle beams tend to be much longer than the plasma wavelength so must be conditioned in order to drive plasma wakefields^22^. Micro-bunching suitably conditions such beams. Micro-bunching is achieved by briefly co-propagating a beam with the focusing and defocusing regions of a high amplitude laser-driven wakefield and then allowing the beam to propagate through vacuum. The beam electrons exposed to the focusing fields drift on-axis, forming the micro-bunches whilst those exposed to the defocusing fields are discarded. This general scheme is demonstrated, via two dimensional particle-in-cell simulations, on the *σ*_*z*_ = 7.68 mm long electron beam generated in the storage ring of the Diamond Light Source (referred to hereafter as the Diamond beam).

**Figure 1:**
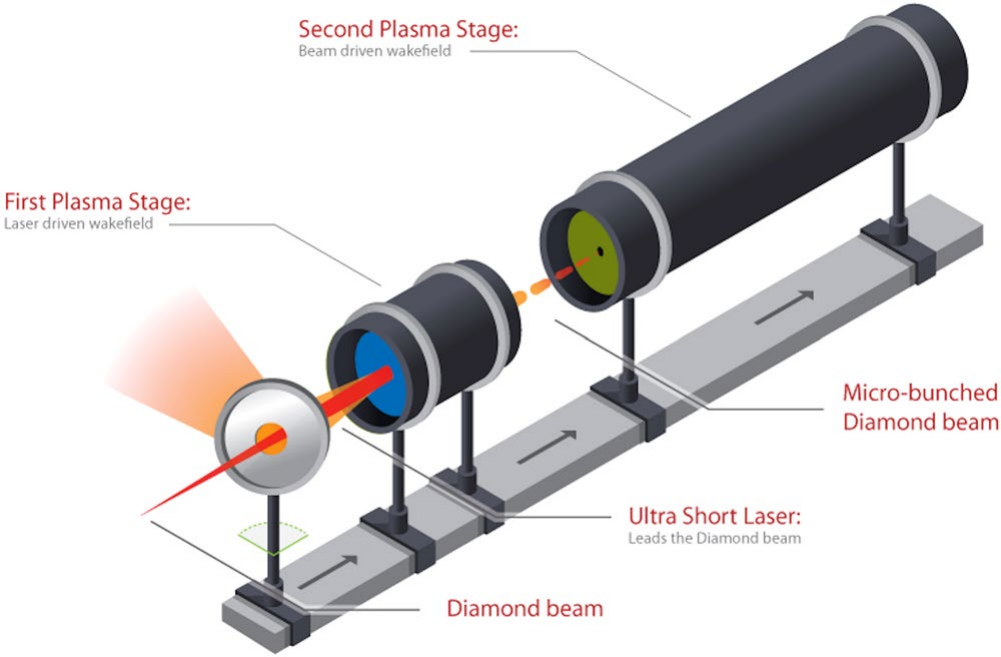
A conceptual design of the two stage plasma cell design. An ultra short laser pulse drives a high amplitude wakefield in the first, short stage that exposes a charged particle beam to alternating focusing and defocusing fields along its length. Micro-bunches form as the beam propagates between the two stages. Upon entering the second stage the micro-bunches resonantly drive a wakefield. This wakefield provides the focusing fields that stimulate whole micro-bunch oscillations.

Co-propagating a particle beam with a laser-driven wakefield throughout the micro-bunch forming process imparts too much transverse momentum to the electrons, causing them to overshoot the beam axis and disperse the would be micro-bunch. Introducing a vacuum gap (Fig. 1), whilst the micro-bunches form, results in a factor of fifty enhancement to the on-axis number density after two metres of propagation when compared to the single stage simulations.

**Figure 2:**
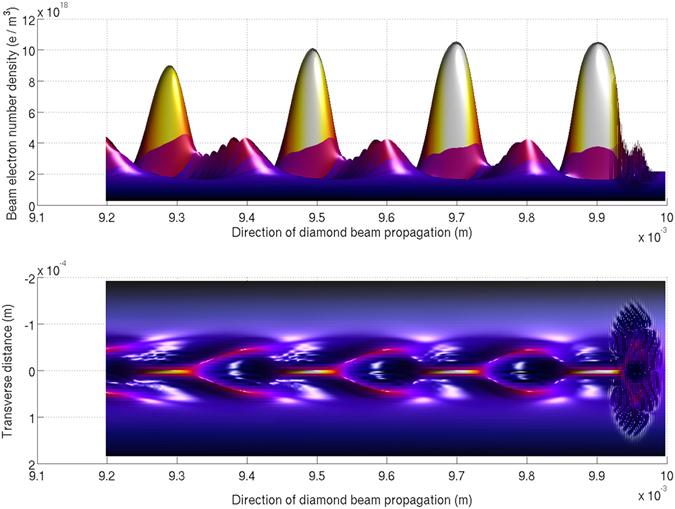
The micro-bunched Diamond beam. Between the yellow micro-bunches are the beam electrons that propagated with the de-focusing regions of the wakefield and are in the process of being transversely expelled. The disturbance to the beam in the right of the panes is due to the laser pulse.

**Figure 3:**
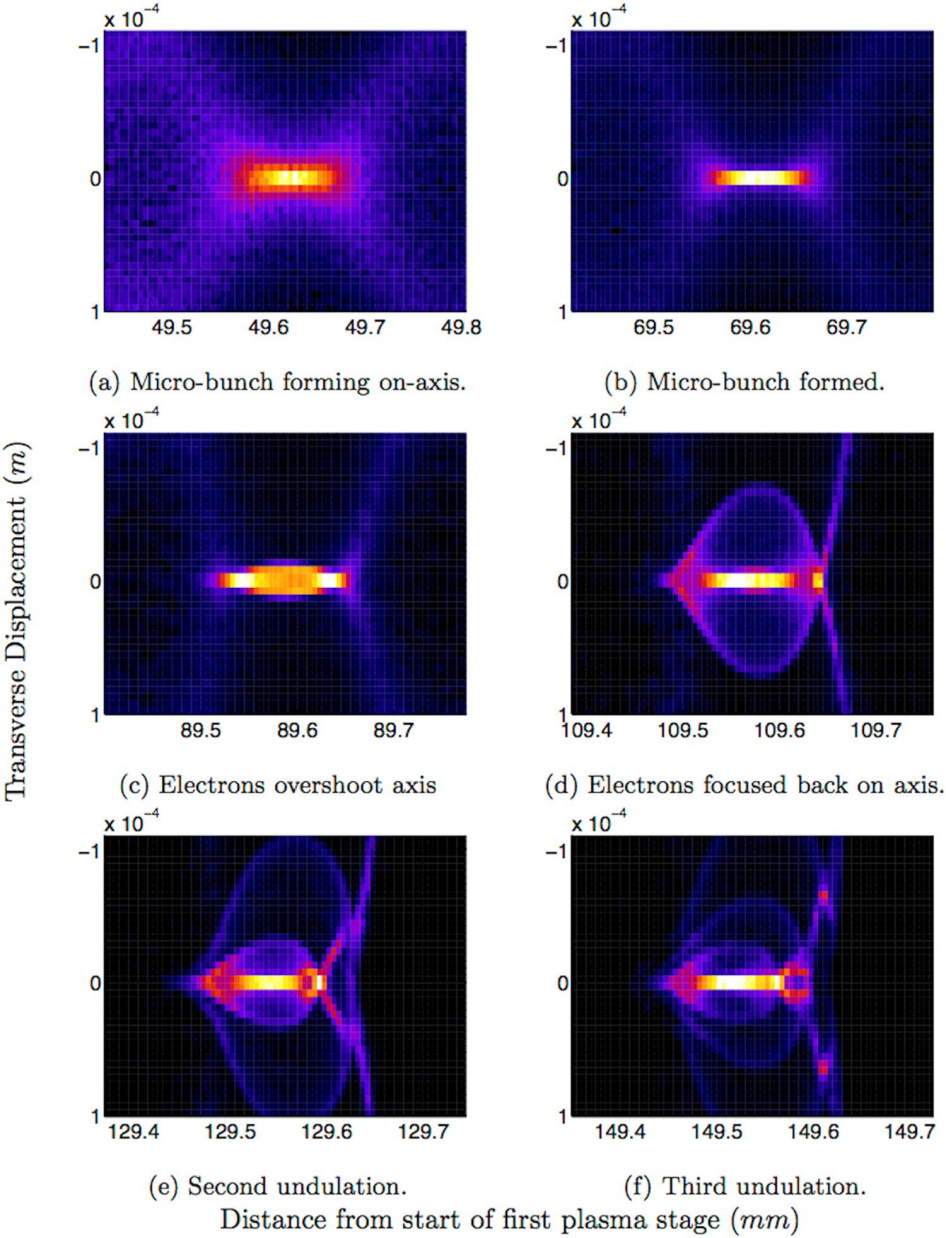
Coherent undulations of the simplified Diamond beam in the second plasma stage. Electron number density of the beam electrons is seen form a top down view. The focusing fields of the wakefield bring the micro-bunch electrons on-axis (**a**). The micro-bunch has maximum on-axis number density in (**b**) and is regarded as formed. The electrons overshoot the axis in (**c**). The bulk of the electrons are focused back on axis whilst some escape the focusing field in (**d**). A second and third undulation is seen in (**e**,**f**).

The *L* = 1 mm first stage has a high amplitude (*E* = 1.3 GVm^−1^) laser-driven wakefield co-propagating with the electron beam. The Diamond beam then micro-bunches over the 114 mm of vacuum, increasing its on-axis number density by a factor of five to *n*_*e*_ = 2 × 10^19^ m^−3^. The micro-bunches are equally spaced as a result of the periodicity of the wakefield, which is crucial to driving a high amplitude wakefield. As each micro-bunch enters the second plasma stage it resonantly enhances the wakefield driven by the previous micro-bunch. The second plasma stage’s position is chosen such that the micro-bunches enter with maximum on-axis charge density (Fig. 2). The micro-bunching process increases the emittance of the beam from 2.84 nm rad to 7.13 nm rad. However, the large focusing fields (*E*_*r*_ = 1.1 GVm^−1^) in this purely particle-driven wakefield keep the micro-bunches contained over the length of the 385 mm second plasma stage (Fig. 3).

The particle-driven wakefield stimulates strong transverse oscillations of the micro-bunches. In contrast to betatron oscillations that arise from electrons self-injected in the blow-out laser wakefield regime (comprising typically of tens of pico-coulombs of charge^23^ oscillating over millimetres), a nano-coulomb of charge in the micro-bunches oscillates over tens of centimetres. These oscillations are about the axis of propagation, and as a consequence, generate radially polarised X-ray pulses. Radially polarised X-rays can overcome the diffraction limit when strongly focused with use of a suitable aperture and have a longitudinal component to the electric field at the focus^24^. These properties could potentially allow for a unique single-atom probe.

X-ray pulses with brilliance comparable to those presented in this paper have been demonstrated experimentally via self-injected electron beams of laser-driven wakefields^25, 26^. Self-injection is a highly non-linear process. Great strides have been made reducing the shot-to-shot variation of the charge, divergence, energy and pointing of the self-injected electron beams, however the variations still remain in the region of 10–15%^27^, far away from the level of control demonstrated by conventional accelerators. The scheme presented here simulates conventionally generated electron beams within plasma wakefields in the quasi-linear regime to produce brilliant, low divergence, radially polarised trains of X-ray pulses providing a unique tool that, if experimentally realised, is believed to be highly reproducible.

**Figure 4:**
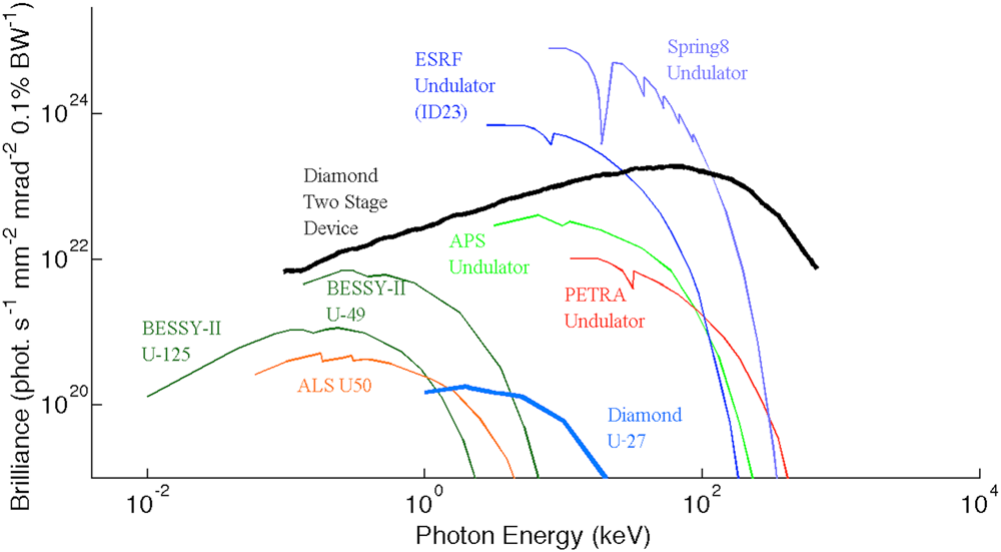
The peak brilliance of various third generation light sources compared to the X-ray pulse by the simulated Diamond storage ring beam. The lower emittance of 2.84 nm rad and shorter beam length of 7.68 mm of the storage ring beam enables the generation of X-ray pulses with photon energies peaking at 59 keV.

The radiated spectrum of the undulating Diamond beam is calculated using a electron tracking code, RDTX^28^. The peak brilliance (Fig. 4) of the X-ray pulse is $\hat{B}$ = 1.9 × 10^23^ photons/(mm^2^ mrad^2^ s 0.1% BW), a three order of magnitude enhancement to that produced by the *L* = 2 m, *B* = 0.79 T, Diamond U-27 undulator ($\hat{B}$ = 1.8 × 10^20^ photons/(mm^2^ mrad^2^ s 0.1% BW)). Equally significant, the brilliance covers a wide range of photon energies, peaking at *E* = 59 keV, which is a factor of 30 higher than the U-27 produced X-ray pulse of *E* = 2 keV.

The train of electron bunches forms a train of X-ray pulses, each 40 *μ*m long with a 200 *μ*m spacing. Furthermore, the X-ray pulses have higher photon frequencies further back in the pulse train as they are generated from micro-bunches experiencing a higher amplitude wakefield. This unique feature offers interesting diagnostic opportunities. The current world record for fastest image acquisition is 4.4 trillion frames per second^29^ and relies upon stroboscopically acquiring a series of illuminations of the target that are dispersed spatially according to their varying photon frequency. It is believed that such an imaging technique would be well suited to using the train of X-ray pulses in single-shot time-resolved studies. For those applications that require sub femtosecond duration X-ray pulses, the second stage can be used to accelerate a suitably conditioned short witness electron beam to 6 GeV^30^, limited by the transformer ratio of a plasma wakefield. This witness bunch can be injected into a conventional undulator to generate harder X-ray pulses with the required pulse duration.

An optimisation study was performed on this two-stage plasma device, culminating in a full final simulation using the refined parameters. Scans over the ultra short laser driver’s parameters; plasma density, plasma stage length, vacuum gap length and beam width versus temperature were performed to maximise the amplitude of the wakefield. It was found that a wakefield of 1.3 GVm^−1^ driven by a modest laser pulse applied to the Diamond beam over 1 mm of propagation, followed by 114 mm of vacuum, fully micro-bunches the Diamond beam, which then drives a wakefield peaking at 2 GVm^−1^ in the 385 mm long second plasma stage.

A scan over the plasma density reveals that the maximum possible wakefield the modulated Diamond beam can drive is 4 GVm^−1^: above this, the wakefield is disrupted by the motion of the background plasma ions. Although the high electric fields return after the disruption, closer examination reveals that there are no distinct wakefields in which to accelerate charged particles. Ion motion can be suppressed by decreasing the charge to mass ratio of the ion: for this simulation set Xenon was therefore chosen.

It has been demonstrated, via extensive, two dimensional computer simulations, that it is possible to micro-bunch the 3 GeV Diamond beam using a novel wakefield accelerator design. Once formed, the micro-bunches maintain their structure via their self-driven wakefield and undergo entire micro-bunch oscillations, generating brilliant, radially polarised X-ray pulses. These pulses have a three order of magnitude enhancement in peak brilliance and a factor of 30 enhancement in photon energy when compared to those generated from conventional magnetic insertion devices. The new two-stage plasma scheme could be used to accelerate a witness electron beam to greater energies than the drive beam, allowing current light sources to generate harder X-rays from the existing infrastructure. The use of higher plasma densities with shorter initial bunches promises even higher peak brilliance gains in the future. In summary, a two-stage plasma scheme allows existing light source facilities to greatly enhance both the brilliance and energy of X-ray pulses with a single insertion device, offering a diagnostic with unique temporal properties allowing for ultra-fast studies.

## Methods

Designing the two plasma stage beam line required a parameter scan over plasma density of both stages, and the length of the first stage, both of which were run in a modified version of the particle-in-cell code EPOCH^31, 32^ Simulations were initially designed with the longer and higher emittance electron beam form the Diamond booster synchrotron. The aim of the density scan was to identify the density at which the largest amplitude wakefield could be driven by the micro-bunched Diamond booster beam without ion motion disruption. To model a micro-bunched beam a tri-gaussian envelope was assumed with a sinusoidal modulation along its length, described by equation 1:

1 $${N}_{b}(x)=\frac{N}{{\mathrm{(2}\pi )}^{\mathrm{3/2}}{\sigma }_{z}{\sigma }_{y}^{2}}{e}^{-{z}^{2}\mathrm{/2}{\sigma }_{z}^{2}}{e}^{-{y}^{2}\mathrm{/2}{\sigma }_{y}^{2}}(1-\,\cos ({k}_{p}z\mathrm{)/2})$$

Here, *σ*_*z*_ is the beam length, *σ*_*y*_ is the beam width, *N* is the number of beam electrons and *k*_*p*_ is the plasma wavenumber. The wakefield amplitude as a function of distance from the head of the Diamond booster beam (+3*σ*_*z*_) is plotted in supplementary figure M0. The density scan identified a density of interest *n*_*e*_ = 2.8 × 10^22^ m^−3^. At this density a wakefield is driven along the length of the Diamond booster beam peaking at 2 GVm^−1^ toward the tail. The artificial micro-bunch train simulated is significantly closer to ideal than the train formed in the final simulation, which achieves a wakefield amplitude of *E* = 1.1 GVm^−1^. The parameters for the density scan are summarised in Tables 1 and 2.

**Table 1:** Diamond beam parameters.

*E* (GeV)	3
Δ*E*/*E*	0.007
$\epsilon$ (nm rad)	140
*σ*_*z*_ (mm)	26
*σ*_*r*_ (m)	$\sqrt{2}/{\kappa }_{p}$
*N*	1.25 × 10^10^

*E* is the electron energy, Δ*E*/*E* is the longitudinal energy spread, $\epsilon$ is the emittance, *σ*_*z*_ is the beam length, *σ*_*r*_ is the beam radius and *N* is the number of electrons.

**Table 2:** Technical and plasma parameters.

Technical	Parameters
*n*_*z*_ (cells)	150
*n*_*y*_ (cells)	150
*z*_*max*_ − *z*_*min*_ (m)	2*λ*_*p*_
*y*_*max*_ − *y*_*min*_ (m)	2*λ*_*p*_
*t*_*end*_ (s)	6*σ*_*z*_/*c*
*ppc*	4
**Plasma**	**Parameters**
*n*_*e*_ (m^−3^)	0.2775 × 10^20^ × 2^*m*^
*m*	5:15
Ion	*Xe* ^+^

*n*_*z*_ is the grid cell length, *n*_*y*_ is the grid cell width, *z*_*max*_ − *z*_*min*_ is the grid length, *y*_*max*_ − *y*_*min*_ is the grid width, *t*_*end*_ is the run time, *ppc* is the particles per cell, *n*_*e*_ is the electron number density, *m* is the variable scanned over and Ion is the plasma ion species.

The aim of the plasma stage length scan was to identify the co-propagation length needed between the Diamond booster beam and the laser-driven wakefield that results in the formation of low emittance micro-bunches after further propagation through vacuum. A laser pulse that drives a *E*_*zmax*_ = 1.3 GVm^−1^ in the plasma density of interest is summarised in Table 3. Resolving the laser wavelength within a particle-in-cell simulation requires approximately two orders of magnitude more computational resources to compute. As such, a way to model the laser pulse was required. An ultra-short electron beam was picked that drove a *E*_*zmax*_ = 1.3 GVm^−1^ wakefield and used to model the laser pulse. For this scan a lower energy *E* = 300 MeV Diamond booster beam was used to further save on runtime. This ultra-short electron beam led the core of the Diamond booster beam by +1*σ*_*z*_, driving a wakefield in the plasma which co-propagates with the trailing Diamond booster beam. The Diamond booster beam then leaves the first plasma stage and forms into micro-bunches as it propagates through vacuum. The distance from the first stage to the point at which the on-axis number density of the forming micro-bunches is maximised is said to be the ‘focal point’ of the first stage. The results are summarised in Table 4. Further simulations set the first plasma cell length to *L*_1*st*_ = 1 mm, yielding micro-bunches with emittance of $\epsilon =7.13\,{\rm{nm}}\,{\rm{mrad}}$ — an acceptable increase of a factor 2.5.

**Table 3:** Laser parameters.

*E* (J)	0.5
*τ* (fs)	50
*σ*_*r*_ (*μ*m)	$\sqrt{2}/{\kappa }_{p}$
*λ* (*μ*m)	1.06
*P* (TW)	10

*E* is the laser pulse energy, *τ* is the beam length, *σ*_*r*_ the beam width, *λ* is the laser wavelength and *P* is the laser peak power.

**Table 4:** First cell focal point.

Cell length (mm)	Focal point (mm)
2.5	65.5
3.0	57.0
3.5	48.5
4.0	42.0
4.5	37.5
5.0	33.0

A full-scale, 2D simulation was then performed of the laser pulse and Diamond booster beam propagating through the first stage, vacuum, and into the second plasma stage. This simulation fully resolved the plasma wavelength capturing the physics neglected in the stage-length scan (Rayleigh length, dephasing length, etc.). Parameters for this simulation are found in Tables 1, 3 and 5. A further simulation was performed applying the two-stage scheme to the Diamond Light Source’s storage ring electron beam. The parameters are the same as those found in Table 5, but with a lower emittance of 2.84 nm rad and shorter beam length of 7.68 mm.

**Table 5:** Full final simulation parameters.

Technical	Parameters
*n*_*z*_ (cells)	1,200,000
*n*_*y*_ (cells)	78
*z*_*max*_ − *z*_*min*_ (mm)	40
*y*_*max*_ − *y*_*min*_ (mm)	2.6 × *λ*_*p*_
*t*_*end*_ (s)	1.666 × 10^−9^
*ppc*	4
**Plasma**	**Parameters**
*n*_*e*_ (m^−3^)	2.84 × 10^22^
*Ion*	*Xe* ^+^

*n*_*z*_ is the grid cell length, *n*_*y*_ is the grid cell width, *z*_*max*_ − *z*_*min*_ is the grid length, *y*_*max*_ − *y*_*min*_ is the grid width, *t*_*end*_ is the run time, *ppc* is the particles per cell and *n*_*e*_ is the electron number density.

To calculate the radiation spectrum emitted by the wiggling Diamond beam the code RDTX was used. Parameters of a representative micro-bunch were taken from the full final simulation at the entry point to the second plasma stage. This bunch was simulated as co-propagating with a wakefield of amplitude *E* = 1.1 GVm^−1^ found in the second stage as simulated in EPOCH. For a single particle the distribution in frequency and angle of energy radiated by an accelerated charge is given by:

2 $$\frac{{d}^{2}I}{d\omega d{\rm{\Omega }}}=\frac{{e}^{2}{\omega }^{2}}{4{\pi }^{2}c}{|{\int }_{-\infty }^{\infty }\hat{{\bf{s}}}\times (\hat{{\bf{s}}}\times \beta ){e}^{i\omega (t-\hat{{\bf{s}}}\cdot {\bf{r}}(t)/c)}{\rm{d}}t|}^{2},$$

where $\hat{{\bf{s}}}$ is in the direction of observation, *ω* is the radiation frequency Ω is the angle of observation from the axis of propagation, *I* is energy, **r** is electron position, *β* = **v**/*c* is the normalised velocity and other symbols have their usual meaning.

The RDTX code calculates the spectral intensity of radiation emitted by a number *N*_*P*_ of accelerating point charges^33^, with the *j*th particle at position *r*_*j*_, and with normalized velocity *β*_**j**_ = **v**_*j*_/*c*, in the far-field, as:

3 $$\frac{{d}^{2}I}{d\omega d{\rm{\Omega }}}=\frac{{\mu }_{0}{e}^{2}c}{16{\pi }^{3}}{\omega }^{2}{|{\int }_{-\infty }^{\infty }\sum _{j=1}^{{N}_{p}}\hat{{\bf{s}}}\times {\beta }_{j}{e}^{i\omega (t-\hat{{\bf{s}}}\cdot {{\bf{r}}}_{{\bf{j}}}/{\bf{c}})}{\rm{d}}t|}^{2}.$$

### Radiated Spectrum of the Oscillating Micro-bunch

The peak brilliance^34^ is simply the number of photons per pulse, per unit volume in six-dimensional phase space, in a frequency window of ±0.0005 *ω* and is given by:

4 $$\hat{B}=\frac{{N}_{B}}{{\mathrm{(2}\pi )}^{3}{\epsilon }_{x}{\epsilon }_{y}{\epsilon }_{E}\tau },$$

where *N*_*B*_ is the number of photons per pulse per 0.1% band width, ${\epsilon }_{x}$ and ${\epsilon }_{y}$ are the transverse rms emittance of the X-ray pulse, ${\epsilon }_{E}$ is the fractional energy spread of the X-ray pulse and *τ* is the length of the X-ray pulse. This calculation can be broken down by first calculating the phase space volume, and then the number of photons per 0.1% frequency window.

### Six Dimensional Phase Space Volume

**Figure 5:**
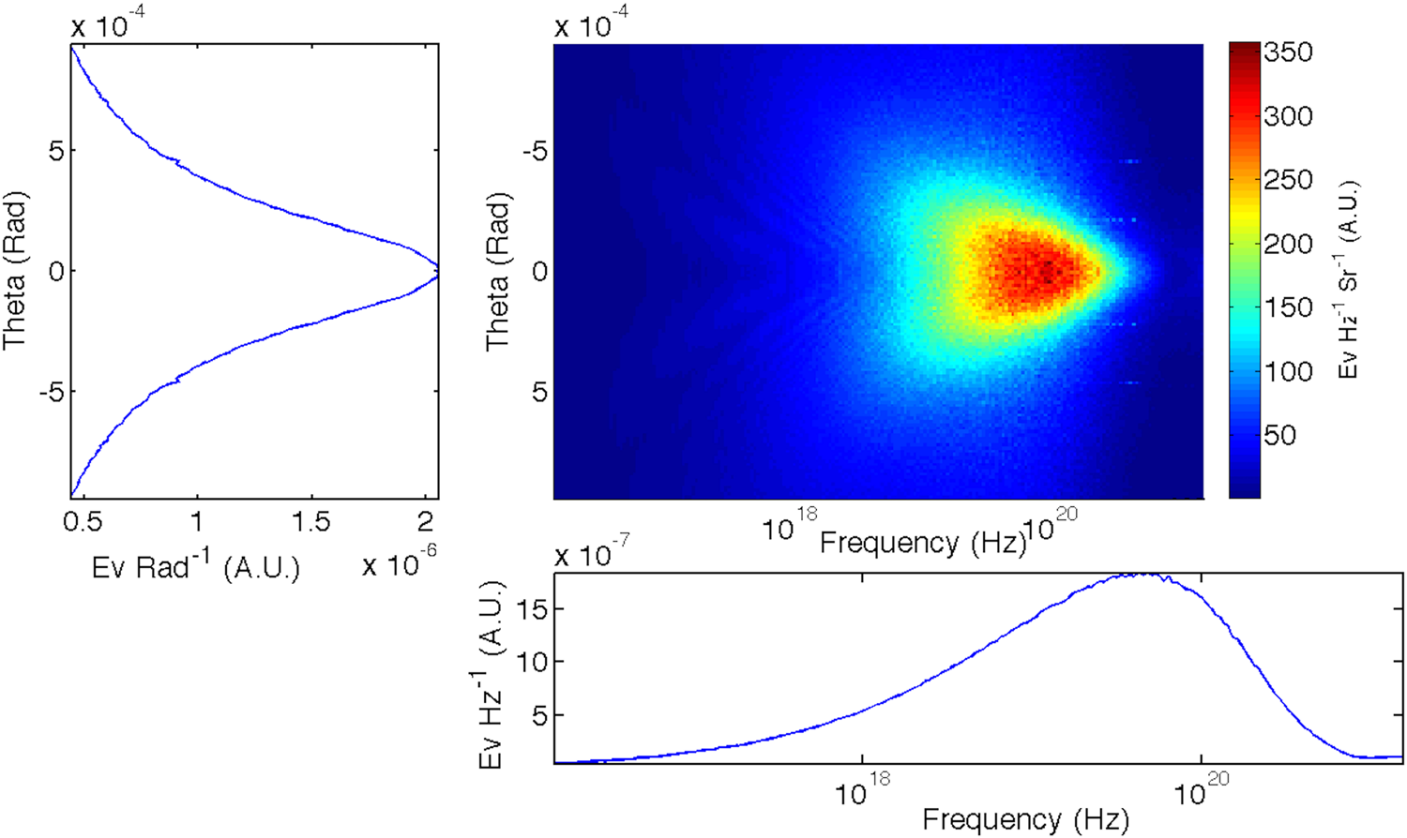
The angularly resolved spectrum emitted by the representative Diamond micro-bunch (top right pane). The left pane is integrated over the frequency of the photons and gives the root-mean-squared divergence of the photons. The bottom pane is integrated over solid angle and gives the spectrum of the radiation.

Figure 5 shows the output of the RDTX code for the Diamond micro-bunch simulation, which is the energy deposited on the virtual spectrometer, as a function of angle from the propagation axis, *θ*, and the frequency of radiation, *ω*. The figure shows that the majority of the energy deposited is close to the axis (*θ* < 0.001 rads) and the spectrum is that of a synchrotron, i.e. it lacks the narrow peaks emitted by an undulator. The left pane is integrated over the frequency of the photons and gives the root-mean-squared divergence of the photons. The bottom pane is integrated over angle of emittance and gives the spectrum of the radiation. The critical frequency of the synchrotron-like spectrum is *ω*_*crit*_ = 4.5 × 10^19^ rad s^−1^ with a FWHM frequency spread of Δ*ω* = 2.1 × 10^20^ rad s^−1^. The longitudinal emittance is found to be ${\epsilon }_{E}={\rm{\Delta }}E/E=2.81$.

**Figure 6:**
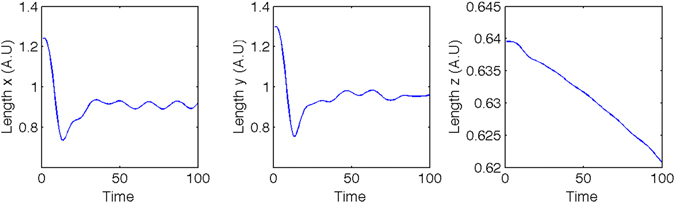
The transverse and longitudinal root-mean-squared beam size of the Diamond micro-bunch in the second plasma stage. The initial transverse size compresses significantly during the first betatron oscillation before undergoing smaller amplitude oscillations. The length of the micro-bunch decreases throughout the simulation as the head of the bunch experiences the decelerating regions of the wakefield and the rear the accelerating.

The transverse size of the particle beam is used as an approximation to the X-ray transverse beam size and is displayed, as a function of time, in Fig. 6. The rms transverse beam size was found to be 〈*x*〉 = 〈*y*〉 = 23 *μ*m. Given the quantities above, the six-dimensional phase space, multiplied by (2*π*)^3^ *τ* to complete the denominator of equation 4, is found to be ${\mathrm{(2}\pi )}^{3}{\epsilon }_{x}{\epsilon }_{y}{\epsilon }_{E}\tau =4.71\times {10}^{-28}\,{{\rm{m}}}^{2}\,{{\rm{rad}}}^{2}\,{\rm{s}}$.

The number of photons per 0.1% bandwidth is found by taking the solid angle integrated spectrum and performing a moving point integration over the frequency window *ω* − Δ*ω* to *ω* + Δ*ω*, where Δ*ω* is 0.005. The peak brilliance is then this quantity divided by the calculated phase space volume and the beam length, *τ*.

The code RDTX calculates the spectral intensity, $\frac{{d}^{2}I}{d\omega d{\rm{\Omega }}}$, emitted by accelerating charge. To calculate the brilliance from this quantity one first finds the number of photons per relative bandwidth per steradian,

5 $$\frac{{d}^{2}I}{d\omega d{\rm{\Omega }}}=\frac{{d}^{2}N\hslash \omega }{d\omega d{\rm{\Omega }}}=\hslash \frac{{d}^{2}N}{\tfrac{d\omega }{\omega }d{\rm{\Omega }}},$$

where N is the number of photons and *ħ* is Planck’s constant. Convention states that peak brilliance is given as the number of photons per 0.1% bandwidth, i.e. $\frac{d}{\tfrac{d\omega }{\omega }}={10}^{3}\frac{d}{d\mathrm{(0.1 \% }\,{\rm{BW}})}$. Furthermore peak brilliance is measured in milliradians squared, so $\frac{d}{d{\rm{\Omega }}}={10}^{6}\frac{d}{d(\pi {\theta }^{2})}$. Substituting these unit conversions into equation 5 yields,

6 $$\frac{{d}^{2}N}{d(\mathrm{0.1 \% }BW)d\pi {\theta }^{2}}=\frac{1}{{10}^{9}\hslash }\frac{{d}^{2}I}{d\omega d{\rm{\Omega }}}.$$

This quantity is the number of photons per 0.1% bandwidth per milliradian squared. To find the peak brilliance one has to divide by the intrinsic source properties of the electron beam generating the pulse, the duration, *τ*, and the source area, ${\sigma }_{r}^{2}$, measured in millimetres squared (this convention adds a factor of 10^6^ to the co-efficient to the right have side of equation 7).

7 $$\hat{B}[\frac{{\rm{photons}}}{{\rm{s}}\,{{\rm{mm}}}^{2}\,{{\rm{mrad}}}^{2}\,0.1 \% \text{BW}}]=\frac{1}{{10}^{15}\hslash {\sigma }_{r}^{2}\tau }\frac{{d}^{2}I}{d\omega d{\rm{\Omega }}}$$

Figure 4 plots the peak brilliance of the single representative micro-bunch against existing third generation light sources. The peak brilliance of the micro-bunch is found using equation 7. There is a three order of magnitude enhancement to peak brilliance when compared to the third generation light sources which extends well into the hard X-ray regime. Broadband emission is seen and there are no characteristic narrow energy bands, as would be expected from an undulator. In contrast to betatron oscillations that arise from electrons injected in the blow-out laser wakefield regime (comprising typically of tens of pico-coulombs of charge oscillating over millimetres), a nano-coulomb of charge in the micro-bunches undergo these coherent oscillations over tens of centimetres. Note that, since the peak brilliance of a synchrotron-like source is proportional to the charge of the beam and inversely proportional to the length of the beam, the peak brilliance of the micro-bunch train will be comparable to the single representative micro-bunch.

## Electronic supplementary material

Supplementary Information
