# Supplementary material for: Brilliant X-rays using a Two-Stage Plasma Insertion Device
Source: Sci Rep. 2017 Jun 21;7:3985. doi: 10.1038/s41598-017-04124-7 (PMC5479796; doi:10.1038/s41598-017-04124-7)
Supplement: Supplementary file 1 — Supplementary Information [file 41598_2017_4124_MOESM1_ESM.pdf]

Brilliant X-rays using a Two-Stage Plasma Insertion Device

J. A. Holloway[1,2,4], P. A. Norreys[3,4], A. G. R. Thomas[6], R. Bartolini[2,5] R. Bingham[4,7], J. Nydell[1], R. M. G. M. Trines[4], R. Walker[5,2] & M. Wing[1]

Correspondence to james.holloway@physics.ox.ac.uk

[1]*Department of Physics and Astronomy, University College London, London, WC1E 6BT, United Kingdom*

[2]*John Adams Institute, University of Oxford, Denys Wilkinson Building, Keble Road, Oxford, OX1 3RH, United Kingdom*

[3]*Department of Physics, University of Oxford, Parks Road, Oxford, OX1 3PU, United Kingdom*

[4]*Central Laser Facility, Rutherford Appleton Laboratory, OX11 0QX, United Kingdom*

[5]*Diamond Light Source, Harwell Science and Innovation Campus, OX11 0DE, United Kingdom*

[6]*Department of Nuclear Engineering & Radiological Sciences, University of Michigan, Ann Arbor, MI, United States*

[7]*Department of Physics, University of Strathclyde, Strathclyde, G4 0NG, United Kingdom*

Figure M0. The wakefield amplitude as a function of distance from the head of the micro-bunched Diamond booster beam ($+3\ \sigma_z$) for four different plasma densities.

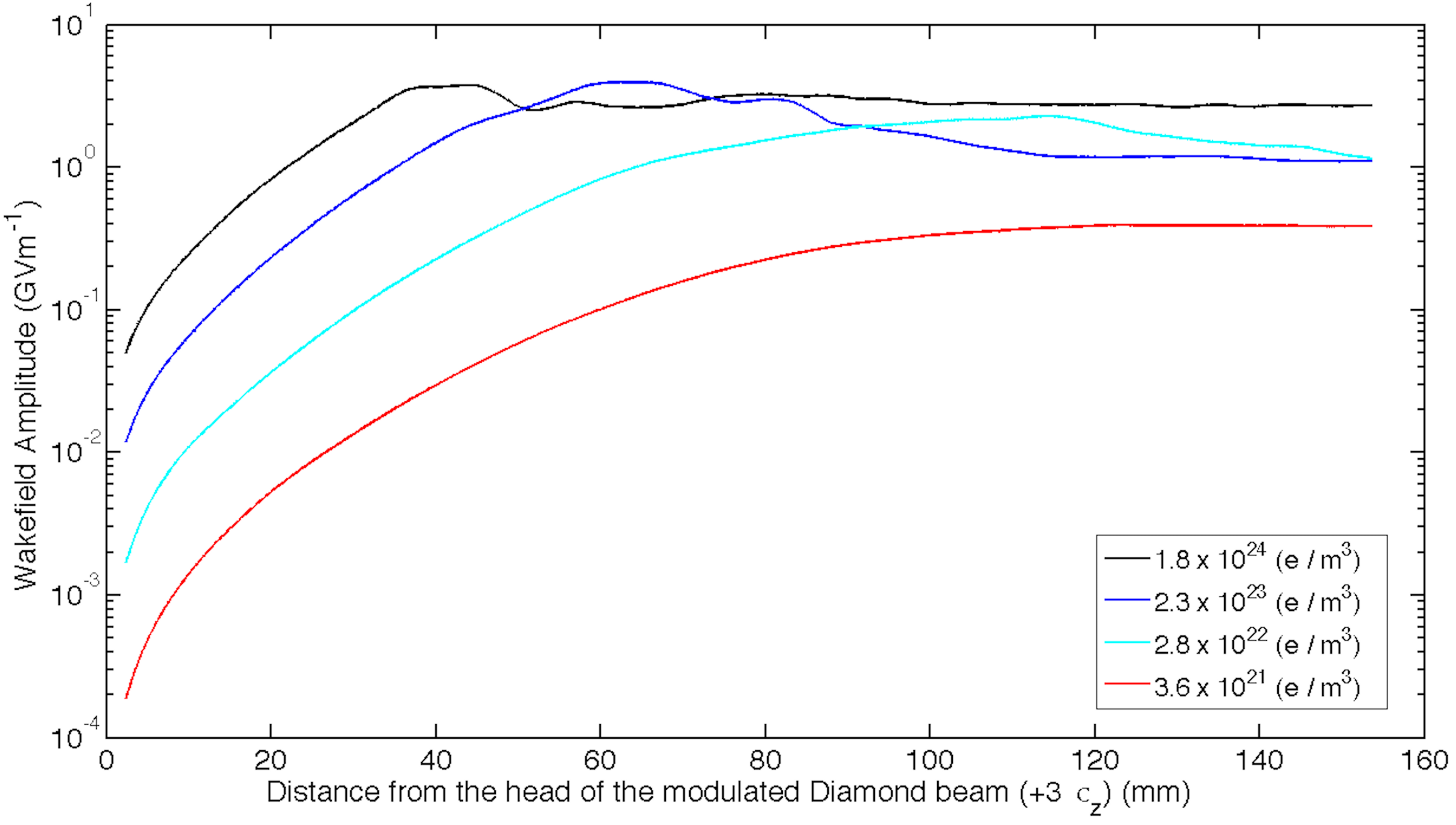

Wakefield Amplitude (GVm^{-1})
10^{1}
10^{0}
10^{-1}
10^{-2}
10^{-3}
10^{-4}
0
20
40
60
80
100
120
140
160
Distance from the head of the modulated Diamond beam (+3 σ_z) (mm)
1.8 x 10^{24} (e / m^3)
2.3 x 10^{23} (e / m^3)
2.8 x 10^{22} (e / m^3)
3.6 x 10^{21} (e / m^3)
